# Self-Efficacy Among Adolescents With Sickle Cell Disease in a Central Indian Tertiary Care Hospital

**DOI:** 10.7759/cureus.77394

**Published:** 2025-01-13

**Authors:** Joyce Joseph, Asha P Shetty, Suganya P

**Affiliations:** 1 College of Nursing, All India Institute of Medical Sciences Raipur, Raipur, IND; 2 College of Nursing, All India Institute of Medical Sciences Bhubaneswar, Bhubaneswar, IND

**Keywords:** adolescents, anemia, self-efficacy, sickle cell, sickle cell disease

## Abstract

Aim: Sickle cell disease (SCD) is a major public health concern. Self-efficacy is a person’s particular set of beliefs in their ability to accomplish daily life activities with symptom management. The aim of the present study was to explore the level of self-efficacy among adolescents with SCD.

Methods: A descriptive cross-sectional study was conducted to assess the self-efficacy of adolescents with sickle cell disease using a purposive sampling of 300 adolescents diagnosed with SCD in a tertiary care center in east-central India. A sickle cell self-efficacy scale was used to collect data between November 2023 and April 2024. Data was analyzed using descriptive statistics and linear regression.

Results: The mean age at which adolescents were diagnosed with SCD was 3.31 ± 0.14 years, and 69.7% (209) of adolescents were identified between the ages of one and five years. Additionally, 73% (219) of adolescents had moderate self-efficacy. The current study found that most adolescents had a moderate level of self-efficacy, and regression analysis showed that the strong predictors for higher self-efficacy levels were higher education, economic status, place of residence, and age group.

Conclusions: The adolescents had a moderate level of self-efficacy. Future interventions are recommended to help improve self-efficacy in adolescents with SCD.

## Introduction

World Health Organization (WHO) recognizes sickle cell disease (SCD) as a main concern for public health. SCD is a common inherited disease that affects more than five million people throughout the world, and approximately 1.2 million people in India are affected by SCD. It was reported that by 2050, there will be a 30% increase in the number of people with SCD. India has been the second highest country for the predicted SCD births, among the world's population. SCD is more prevalent among many tribal populations of India, mainly in the states of Madhya Pradesh, Odisha, and Chhattisgarh [1,2]. A recent field study report showed at least one or at most three SCD patients per 1,000 population in Chhattisgarh [3]. The prevalence of the sickle cell allele in the Chhattisgarh population is nearly 10% during initial screenings, and the rate is as high as 30% in some communities [4].

SCD is a lifelong inherited disorder that can cause many complications throughout an individual’s life. Much of the care plan for this disease is interwoven with daily life behaviors, so SCD individuals are the most responsible for the control and management of the disease [5,6]. A better understanding of self-care can help healthcare providers equip patients with the resources and skills necessary to participate in their disease management [7].

Self-efficacy is a person’s particular set of beliefs in their ability to accomplish daily life activities with symptom management [8]. Assessing self-efficacy helps a lot to decrease the burden on the family, promote self-confidence, and ensure continued care, ultimately producing more beneficial outcomes in the care plan. Moreover, it will enable the patients, families, and communities to take initiative and assume responsibility for the effective development of their own care toward improving the quality of life, health, and well-being.

Self-efficacy can help individuals feel more confident in mastering challenging situations and develop a strong sense of commitment to targeted tasks, ultimately leading to positive health behavior changes. Efficacy beliefs are one attribute of ownership of health and are essential for mastering health behaviors [9]. This study aims to assess the level of self-efficacy and identify the predictors associated with self-efficacy among adolescents with SCD in central India.

## Materials and methods

Study design and participants

This descriptive cross-sectional study enrolled adolescent children (10-19 years) diagnosed with SCD who were attending the special sickle cell clinic every Thursday and those admitted in the pediatric medical wards of a tertiary care teaching hospital in Raipur, Chhattisgarh, India. This study was conducted over a six-month period from November 2023 to April 2024.

The inclusion criteria were adolescent children (10-19 years) with a confirmed diagnosis of SCD, visiting the tertiary care hospital during the study period, and willing to participate in the study. The study excluded adolescents with SCD who were unconscious or critically ill.

Data collection

A semi-structured questionnaire was used to collect demographic details, and the self-efficacy levels were measured through the sickle cell self-efficacy scale, as mentioned below.

Demographic performa questionnaire

A self-structured demographic performa questionnaire was used to collect the demographic and disease profiles of study participants. It includes details such as age, gender, religion, area of residence, education of the child, type of family, number of siblings, birth order of the affected child, socioeconomic status, age at which SCD was diagnosed, self-reported symptoms, complications, and healthcare accessibility (Table 1).

**Table 1:** Tool 1 (demographic performa)

Demographic variables	
Age of the child (in years)	10-12
	13-14
	15-16
	17-19
Gender of the child	Male
	Female
Education of the child	Primary education
	Secondary education
	Higher secondary
	Graduation and above
Area of residence	Urban
	Rural
Type of family	Single parent family
	Nuclear family
	Extended family
Number of siblings	≤2
	>2
Birth order of the affected child	First child
	Second child
	Third or above
Socioeconomic status	Lower
	Lower middle
	Upper lower
	Upper middle
	Upper
Any relative affected by sickle cell disease	No
	One relative
	2 or more
Clinical profile	
Age at which sickle cell disease diagnosed	Infancy (0-12 months)
	Childhood (1-5 years)
	Adolescence (6-19 years)
Self-reported symptoms	Absent
	Present. If present, what are the symptoms?
Complications	Present. If present, what are the complications?
	Absent
Healthcare accessibility	Primary Health Center
	Community Health Center
	Medical College
	Private
Number of hospital admissions (yearly)	No admission
	One time
	>2 times

Sickle cell self-efficacy scale (SCSES)

The SCSES was developed by Edwards et al. [10] as a valid and reliable scale (2000), and it was used to measure disease-specific perceptions of self-efficacy, comprising nine questions. A five-point Likert scale is used to measure the responses, ranging from 1 (not at all) to 5 (completely sure). The scores are categorized into three levels: low (9-20.99), moderate (21-32.99), and high (33-45). The higher scores indicate a more optimistic attitude and more self-efficacy. The scale was translated into Hindi also and found to be reliable. Test-retest reliability was done using the Karl-Pearson correlation coefficient (0.78).

Sample size and sampling strategy

Purposive sampling was conducted in this study, and the sampling bias was reduced by enrolling participants who fulfilled the inclusion criteria. The sample size was calculated using the Cochran formula:

$n = Z^2P\frac{(1-P)}{d^2}$

where Z = 1.96 for a 95% confidence level, d = margin of error (0.05), and P = 0.23 (estimated prevalence from a previous study) [11]. The sample size calculated was 276. After adjusting for a 10% non-response rate, the final sample size was determined to be 300.

​Statistical analysis

The data were analyzed using the Statistical Package for the Social Sciences (SPSS), version 26 (IBM Corp., Armonk, NY). Descriptive statistics were applied. The categorical variables were presented as counts and percentages. The linear regression model was performed to assess the predictors of self-efficacy among adolescents with SCD. P-value < 0.05 was considered statistically significant.

## Results

Three hundred adolescents with SCD (10-19 years old) were included in this study. Among them, 189 (63%) had attained secondary education, and 153 (51%) were female. Of the adolescents, 46.7% (140) came from rural areas. Regarding the type of family, almost 144 (48%) came from nuclear households, and 146 (48.7%) came from extended families. In the birth order, more than half of the adolescents, 164 (54.7%), were firstborns. In terms of socioeconomic status, more than one-third of the adolescents ​​​​​belonged to the middle class, with 94 (31.3%) in the upper middle class and 149 (49.7%) in the lower-middle class, ​respectively. Regarding SCD in the family history, 64.3% (193) of adolescents had a single relative, and 21% (63) had two or more relatives with SCD (Table 2).

**Table 2:** Demographic and clinical profile of adolescents with sickle cell disease (n = 300) SCD: Sickle cell disease; PHC: Primary Health Center; CHC: Community Health Center.

Demographic profile		n	%
Age	10-12 years	68	22.7
	13-14 years	78	26.0
	15-16 years	80	26.7
	17-19 years	74	24.7
Sex	Male	147	49.0
	Female	153	51.0
Education	Primary education	44	14.7
	Secondary education	189	63.0
	Higher secondary	51	17.0
	Graduation and above	16	5.3
Religion	Hindu	294	98.0
	Muslim	6	2.0
Place of residence	Urban	160	53.3
	Rural	140	46.7
Type of family	Single parent family	10	3.3
	Nuclear family	144	48.0
	Extended family	146	48.7
No. of siblings	≤2	213	71.0
	>2	87	29.0
Birth order of the affected child	First child	164	54.7
	Second child	108	36.0
	Third or above	28	28.0
Socioeconomic status	Lower	7	2.3
	Lower middle	149	49.7
	Upper lower	39	13.0
	Upper middle	94	31.3
	Upper	11	3.7
Any relative affected by sickle cell disease	No	44	14.7
	One relative	193	64.3
	>2 or more	63	21.0
Clinical profile		n	f
Age at which SCD was diagnosed	Infancy (0-12 months)	51	17.0
	Childhood (1-5 years)	209	69.7
	Adolescence (6-18 years)	40	13.3
Self-reported symptoms	Absent	41	13.7
	Present	259	86.3
Complications	Absent	168	56.0
	Present	132	44.0
Healthcare accessibility	PHC	95	31.7
	CHC	53	17.7
	Medical College	140	46.7
	Private	12	4.0
No. of hospital admissions (yearly)	No admission	33	11.0
	One time	159	53.0
	>2 or more times	108	36.0

**Figure 1:**
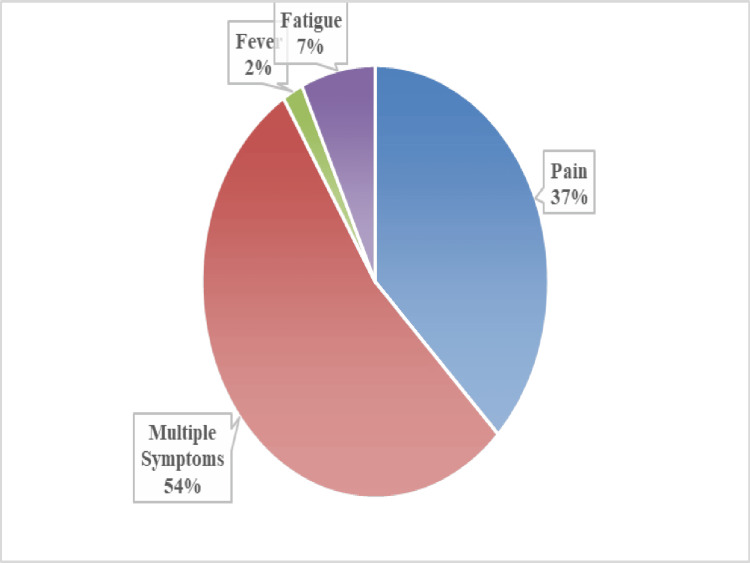
Self-reported symptoms among adolescents with sickle cell disease (n = 259)

The mean age of SCD diagnosed among adolescents was 3.31 ± 0.14 years, and 209 (69.7%) of them were identified between one and five years. Above three-fourth, 259 (86.3%), of the adolescents self-reported their symptoms, and above half of them, 132 (56%), had complications. In terms of SCD treatment, nearly half, 140 (46.7%), of the adolescents received treatment from the medical college, and one-third, 95 (31.7%), received treatment from the primary health center. Among those admitted to the hospital, above half, 159 (53%), of the adolescents had been admitted at least once, and 108 (36%) had been admitted twice or more (Table 2). Among the self-reported symptoms, the majority, 139 (54%), of the adolescents had multiple symptoms, and 97 (37%) had pain (Figure 1).

**Figure 2:**
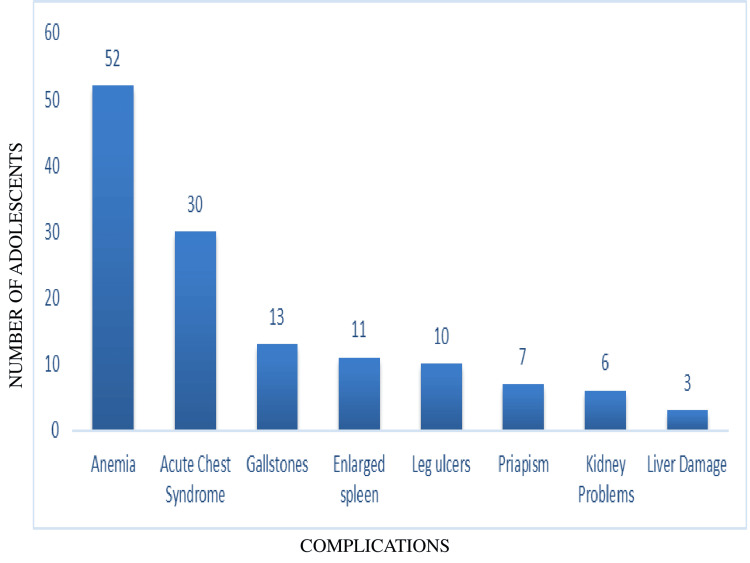
Complications among adolescents with sickle cell disease (n = 132)

In terms of complications, more than half of the adolescents, 168 (56%), did not have any complications. Among the remaining 132 (44%), anemia (54, 18%) and acute chest syndrome (30, 10%) were the most common (Figure 2).

**Figure 3:**
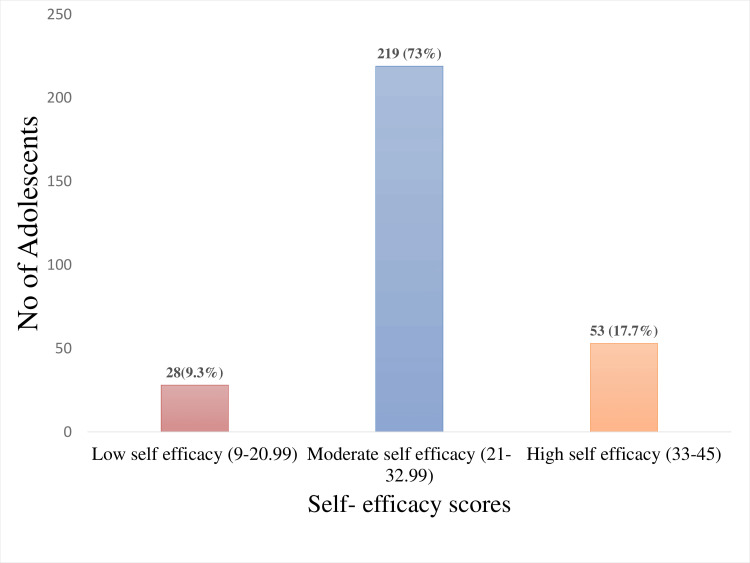
Self-efficacy scores of adolescents with sickle cell disease (n = 300)

The mean self-efficacy score among the adolescents was 24.42 ± 7.04. Approximately three-fourths, 219 (73%), had moderate self-efficacy, 53 (17.7%) had high self-efficacy, and 28 (9.3%) had low self-efficacy (Figure 3).

The regression analysis indicates that adolescents in the higher age group (15-16 years) had a significant association with higher self-efficacy (p < 0.01). Education was a strong predictor of higher self-efficacy, with adolescents having higher education levels showing significantly greater self-efficacy compared to those with only primary education (p < 0.01). Adolescents from rural areas showed significantly higher self-efficacy than those from urban areas (p < 0.05). The socioeconomic status showed that the higher economic group had significantly higher self-efficacy (p < 0.05) than the adolescents in the lower-middle group.

Additionally, adolescents who received treatment from medical colleges (p < 0.01) and private hospitals (p < 0.01) had significantly higher self-efficacy levels than those treated at primary healthcare centers. The number of hospital admissions negatively predicted the self-efficacy level (coef.: -5.41, p < 0.01). Adolescents with frequent hospitalizations had lower self-efficacy scores, indicating that an increased level of hospitalization reduced their self-efficacy level (Table 3).

**Table 3:** Linear regression analysis for the predictors of self-efficacy among adolescents with sickle cell disease (n = 300)

Demographic profile		Self-efficacy level			
		Coefficient	P-value	[95% confidence interval]	
Age	10-12 years	Reference			
	13-14 years	1.84	0.07	-0.17	3.86
	15-16 years	3.06	0.00	1.06	5.07
	17-19 years	0.44	0.67	-1.57	2.45
Sex	Male	Reference			
	Female	-0.36	0.62	-1.76	1.05
Education	Primary education	Reference			
	Secondary education	4.18	0.00	2.14	6.22
	Higher secondary	7.96	0.00	5.41	10.51
	Graduation and above	6.27	0.00	2.76	9.77
Place of residence	Urban	Reference			
	Rural	1.61	0.02	0.23	2.99
Socioeconomic status	Lower	3.31	0.15	-1.23	7.85
	Lower middle	Reference			
	Upper lower	2.56	0.02	0.42	4.69
	Upper middle	1.08	0.18	-0.50	2.67
	Upper	2.31	0.23	-1.50	6.12
Clinical profile					
Self-reported symptoms	Absent	Reference			
	Present	-0.99	0.36	-3.08	1.11
Complications	Absent	Reference			
	Present	-0.88	0.24	-2.34	0.58
Healthcare accessibility	Primary Health Center	Reference			
	Community Health Center	0.59	0.57	-1.45	2.63
	Medical College	3.12	0.00	1.54	4.69
	Private	8.35	0.00	4.77	11.93
Number of hospital admissions (yearly)	No admission	Reference			
	One time	-1.67	0.15	-3.93	0.60
	>2 or more times	-5.41	0.00	-7.76	-3.06

## Discussion

The findings of this descriptive study highlighted the level of self-efficacy in adolescents with SCD in Central India. Studies have highlighted that low self-efficacy level is associated with adverse physical and psychological symptoms, while high self-efficacy leads to positive health outcomes among adolescents [12,13]. Our findings showed that three-fourths of the adolescents, 219 (73%), had moderate self-efficacy, 28 (9.3%) had low self-efficacy, and the remaining had high self-efficacy levels. Among the study participants, more than half were female, and there was an even distribution of SCD across genders, which shows equal inheritance as it is autosomal recessive: 49% (147) were male, and 51% (153) were female. Regarding the level of education, more than half, 189 (63%), of the adolescents had secondary education. The present study showed that SCD was diagnosed before the age of six among adolescents. A study conducted in Chhattisgarh showed that the maximum number of SCD was seen between the ages of one and five years during screening [14]. The current study also indicated that the birth order of the affected children was mostly the first child in the family; this is consistent with another study that testified that 40% of SCD cases occurred in firstborn [15].

Our findings showed that nearly half, 149 (49.7%), of the study participants belonged to the lower-middle class, and 193 (65%) had a family history of SCD in at least one relative. Regarding the admission of pain crisis yearly, 159 (53%) had hospital admission at least once, and pain was the most frequent symptom reported by adolescents. The other symptoms were fatigue, fever, swelling, and multiple other manifestations​​​​​​, with similar findings observed in previous studies ​​​​[16,17]. In the present study, 259 (86.3%) of adolescents were able to self-express their symptoms, and nearly half, 132 (44%), of them presented with complications, which coincided with a recent study showing a high degree of morbidity with complications [18]. Our findings showed that a majority of the adolescents, 219 (73%), reported moderate self-efficacy, which is consistent with several studies [19-21].

The linear regression analysis showed that older age groups, higher levels of education, rural residence, higher economic groups, and receiving treatment from medical colleges or private hospitals are strong predictors for higher self-efficacy among adolescents with SCD. The increased hospital admissions negatively predicted the self-efficacy level.

This study has several limitations. The findings cannot be generalized, as the study did not evaluate patients' self-care in relation to self-efficacy and was conducted in a single tertiary care center, where a large number of SCD patients sought care. Additionally, as a cross-sectional analysis, the study could not establish the directionality of some clinical variables. This study recommends that self-efficacy be routinely measured in clinical practice. Further longitudinal research is needed to explore the extent of self-efficacy level among adolescents over time to better support its clinical relevance.

## Conclusions

In summary, this study found that the majority of adolescents had a moderate level of self-efficacy. Regression analysis showed that the strong predictors of higher self-efficacy levels were higher education, economic status, place of residence, and age group. Self-efficacy is key to guiding self-care interventions, and further strategies and research should be implemented to improve self-efficacy levels among the SCD population.
